# Haemagglutinin displayed on the surface of *Lactococcus lactis* confers broad cross-clade protection against different H5N1 viruses in chickens

**DOI:** 10.1186/s12934-020-01453-7

**Published:** 2020-10-15

**Authors:** Han Lei, Tong Gao, Qianhong Cen, Xiaojue Peng

**Affiliations:** 1 College of Medicine, Southwest Jiaotong University, Chengdu, 610031 Sichuan China; 2 Department of Biotechnology, College of Life Science, Nanchang University, Jiangxi, 330031 China

**Keywords:** *L. lactis*/pNZ8148-spax-HA, Cross protection, H5N1 cross-clade vaccine

## Abstract

**Background:**

The highly pathogenic avian influenza (HPAI) H5N1 virus poses a potential threat to the poultry industry. The currently available avian influenza H5N1 vaccines for poultry are clade-specific. Therefore, an effective vaccine for preventing and controlling H5N1 viruses belonging to different clades needs to be developed.

**Results:**

Recombinant *L. lactis*/pNZ8148-Spax-HA was generated, and the influenza virus haemagglutinin (HA) protein of A/Vietnam/1203/2004 (H5N1) was displayed on the surface of *Lactococcus lactis* (*L. lactis*). Spax was used as an anchor protein. Chickens vaccinated orally with unadjuvanted *L. lactis*/pNZ8148-Spax-HA could produce significant humoral and mucosal responses and neutralizing activities against H5N1 viruses belonging to different clades. Importantly, unadjuvanted *L. lactis*/pNZ8148-Spax-HA conferred cross-clade protection against lethal challenge with different H5N1 viruses in the chicken model.

**Conclusion:**

This study provides insights into the cross-clade protection conferred by unadjuvanted *L. lactis*/pNZ8148-Spax-HA, and the results might help the establishment of a promising platform for the development of a safe and effective H5N1 cross-clade vaccine for poultry.

## Background

Due to its high mortality and antigen drift rate, the highly pathogenic avian influenza (HPAI) H5N1 virus is associated with severe disease and poses a serious threat to the poultry industry [1]. HPAI H5N1 viruses have undergone significant genetic diversification, and to date, 10 viral clades, denoted clades 0 to 9, have been identified. Among these clades, clade 2 exhibits significant genetic variation and has been classified into numerous subclades [2]. Numerous H5N1 influenza vaccines from candidate clade 1 and 2 viruses have been approved for production [3]. In addition, most licensed inactivated and live-attenuated H5N1 vaccines are produced in embryonated chicken eggs infected with the seed viruses, and the manufacturing process can take up 9 months [4, 5]. Unfortunately, the highly diverse genetic nature and the rapid evolution of H5N1 viruses has resulted in titre reduction in high-quality allantoic fluid [6]. Furthermore, the currently licensed H5N1 influenza vaccines and inactivated egg-derived whole-virus H5N1 vaccines provide inadequate immunogenicity against infection with H5N1 belonging to a different clade [7], and the current vaccine manufacturing capacity would be inadequate during an emerging H5N1 pandemic [8, 9]. Because the rapid generation of a well-matched H5 vaccine would represent a challenging task at the onset of a pandemic, two important issues to resolve include the supply of sufficient H5 vaccine doses in a timely manner and evaluating their cross-clade protection efficacy against newly emerged strains of H5 influenza viruses. Therefore, the development of a cross-clade protective vaccine is an ongoing high-priority effort for preparing the domestic poultry industry against a potential HPAI H5N1 epidemic.

Several vaccine approaches have been developed against different H5N1 clades. A monovalent H5 vaccine with RG-epitope-chimeric H5N1 protects mice from lethal challenge with H5N1 viruses of different clades, including clades 1, 2.1, 2.2 and 2.3 [10]. H5N1 VLP vaccines also provides cross-clade protection against both A/Viet Nam/1203/2004 (clade 1) and A/Indonesia/05/2005 (clade 2) [11]. Viable, computationally derived vaccine seed viruses, called the ancestral viruses' vaccines, can be constructed within the context of currently licensed vaccine platforms, and confer protection against morbidity and mortality in ferrets challenged with H5N1 strains from clades 1, 2.1, and 2.2 [12]. Oral administration of encapsulated baculovirus displaying HA (En-BacHA) formulation induces strong cross-clade neutralization against heterologous H5N1 strains (clade 1, clade 2.1, clade 4 and clade 8) and protects mice from challenge with heterologous H5N1 strain (clade 1) [13]. Also, gastrointestinal delivery of live BacHA can provide 100% protection against homologous (clade 2.1) and heterologous (clade 1) H5N1 [14]. Cross-protective immunity study also indicates that mice immunized either orally or subcutaneously with live bivalent-BacHA can be completely protected against clade 1 and clade 2.2.1.1 H5N1 viral infections [15]. Furthermore, an MVAtor vector expressing three H5HA antigens A/Vietnam/1203/04, A/Indonesia/669/06 and A/Anhui/01/05 (MVAtor-tri-HA vector) elicits broad cross-protection against diverse clades by covering amino acid variations in the major neutralizing epitopes of HA among H5N1 subtypes [16]. A vesicular stomatitis virus-based influenza vaccine administered via a single immunization confers rapid protection against different H5N1 clades in a mouse model [17].

It is well -recognized that adjuvants such as AS03 and MF59 have been used in the currently licensed H5N1 vaccine. Prepandemic influenza vaccine H5N1 [Prepandrix(trade mark); AS03-H5N1 vaccine] is approved in the Europe for use as an active immunization against H5N1 subtype influenza A virus (influenza A/H5N1 virus) in adults aged 18–60 years [18]. The antibody-neutralization titres elicited by the FDA-approved AS03-adjuvanted H5N1 are measured gainst H5N1 vaccine strains of A/Vietnam/1194/2004 (clade 1), A/Indonesia/5/2005 (clade 2.1), A/Turkey/15/2006 (clade 2.2), A/Egypt/3072/2010 (clade 2.2), and A/Anhui/1/2005 (clade 2.3.4) as well as the H1N1pdm09 (A/California/7/2009) [19]. In addition, MF59-adjuvanted A/H5N1 vaccine in clinical trials can induce adequate antibody responses against homologous and cross-clade A/H5N1 virus [20–22].

*L. lactis* has been used for the expression of heterologous proteins, such as viral antigens, cytokines and enzymes [23, 24]. Importantly, the *L. lactis* expression system is suitable as a promising vaccine platform for the development of animal influenza A viruses. Our previous studies showed that combined with mucosal adjuvant or within an enteric capsule, *L. lactis* expressing H5N1 HA, HA1 or NP is a safe and effective delivery vehicle against homologous H5N1 virus challenge in a mouse or chicken model [25–27]. Furthermore, recombinant *L. lactis* expressing functional influenza NA or M2e proteins can induce effective mucosal and systemic immune responses in the intestine as well as in the upper respiratory airways (trachea) of chickens, and protect MDCK cells against A/PR/8/34 (H1N1) virus challenge [28]. However, whether unadjuvanted *L. lactis* can provide cross-clade protective immunity against different H5N1 viruses in the chicken model has not yet been investigated.

In the present study, we generated and tested the *L. lactis* delivery vector expressing the H5N1 HA of A/Vietnam/1203/2004 (clade 1) to demonstrate the feasibility of the *L. lactis* display platform for a well-matched H5N1 vaccine. Chickens vaccinated orally with a prime/boost regimen of unadjuvanted *L. lactis* displayed H5N1 vaccine candidates, which could elicit a significant humoral immune response, a significant mucosal immune response, and a neutralizing antibody response. Most importantly, the vaccinated chickens were protected from lethal challenge with different H5N1 clades.

## Methods

### Construction of the *L. lactis* vectored vaccine

**Fig. 1:**
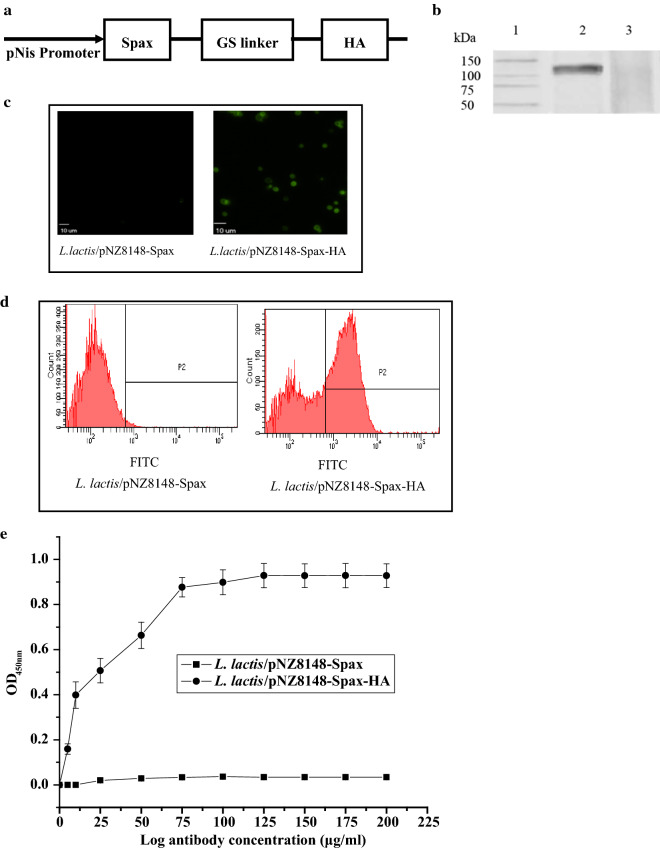
Characterization of the HA protein displayed on the surface of *L. lactis*. **a** Schematic diagram of *L. lactis*/pNZ8148-Spax-HA. A GS linker was inserted between Spax and HA to stabilize the expression of the HA protein. **b** Western blot analysis. Lane 1: Precision Plus Protein™ WesternC™ (Bio-Rad, USA) marker; Lane 2: *L. lactis*/pNZ8148-Spax-HA; Lane 3: *L. lactis*-pNZ8148-Spax. **c** Immunofluorescence microscopy assay of the HA protein: *L. lactis*/pNZ8148-Spax (left) and *L. lactis*/pNZ8148-Spax-HA (right) (magnification: 1,000 ×). **(d)** Flow cytometric analysis of the HA protein displayed on the surface of *L. lactis*: *L. lactis*/pNZ8148-Spax (left) and *L. lactis*/pNZ8148-Spax-HA (positive rate: 60.5%) (right). A total of 15,000 cells were counted. **(e)** Quantification of the HA protein expressed by *L. lactis*/pNZ8148-Spax-HA by indirect ELISA. The values were determined from three independent experiments, and the bars indicate the means ± SDs

The Spax (411 bp) gene was used as an anchor domain and amplified by PCR from the *Staphylococcus aureus* (*S. aureus*) genome as described previously [26]. The HA gene fragment (1650 bp) of A/Vietnam/1203/2004 (H5N1) (GenBank accession No. EU122404) without the signal and transmembrane region was amplified by PCR from pcDNA3.1-HA (kindly provided by Institute of Virology, Chinese Academy of Science, Wuhan, China) using the following primers (the GS linker sequence and *Hind* III are underlined): H-F, 5′ GGCGGCGGCGGCGCCGATCAGATTTGCATTGGTTAC 3′; and H-R, 5′ CCGAAGCTTTTAAATGCAAATTCAGCATT 3′. The Spax and HA fragments were fused into Spax-HA using the primers S-F (5′ CTAGCTAGCAGTCTTCTAACCGAG 3′) and H-R via a GS linker. The resulting Spax-HA containing *Nhe* I/*Hind* III was subcloned into an *L. lactis* expression vector, pNZ8148 (Fig. 1a), and then electroporated into competent *L. lactis* NZ9000. The positive clone of *L. lactis*/pNZ8148-Spax-HA was screened and expressed as described previously [26]. *L. lactis*/pNZ8148-Spax was used as a negative control for the subsequent analyses.

### Nisin-controlled induction of the *L. lactis* vectored vaccine

*Lactococcus lactis*/pNZ8148-Spax and *L. lactis*/pNZ8148-Spax-HA cells were individually cultured in M17 broth medium with 10 μg/ml chloramphenicol and 0.5% (wt/vol) glucose (GM17) at 30 °C overnight without agitation. 1:25 diluted overnight cultures of *L. lactis*/pNZ8148-Spax or *L. lactis*/pNZ8148-Spax-HA cells were transferred into the fresh GM17 medium, respectively. When an optical density (OD) at 600 nm (OD_600nm_) reached 0.3 to 0.4, nisin A was added at a final concentration of 10 ng/ml, and then continued to culture for 3 h at 30 °C without agitation.

### Western bolt analysis

The HA protein expression level in recombinant *L. lactis* was determined by Western blot analysis as described previously [25]. Briefly, 10^8^ cells of *L. lactis*/pNZ8148-Spax-HA pellets were washed three times with 500 µL of sterile phosphate-buffered saline (PBS), resuspended in 50 µL of 6× loading buffer and boiled for 10 min. The treated samples were subjected to SDS–polyacrylamide gel electrophoresis and then transferred to nitrocellulose membranes (Bio-Rad, Hercules, CA, USA). After blocking with 5% non-fat milk at room temperature for 2 h, the membrane was incubated with a monoclonal mouse anti-HA antibody overnight at 4 ℃ and then with affinity-purified horseradish peroxidase (HRP)-conjugated anti-mouse IgG (Sigma-Aldrich Corporation, St. Louis, MO, USA). The membrane was subsequently reacted with the West Pico Chemiluminescent Substrate (Thermo Fisher Scientific Inc., Rockford, IL, USA) and imaged using the Molecular Imager ChemiDoc XRS system (Bio-Rad Laboratories, Inc., Hercules, CA, USA). Meanwhile, Precision Plus Protein™ WesternC™ (Bio-Rad, USA) was served ed as a protein marker.

### Immunofluorescence assay and flow cytometry analysis

The display of HA protein on the surface of *L. lactis* was confirmed by immunofluorescence assay (Olympus IX70, Japan) and flow cytometry (FACS) analysis (BD FACS Calibur, BD Bioscience, San Jose, CA, USA). Briefly, 10^8^ cells of *L. lactis*/pNZ8148-Spax-HA were washed three times with sterile PBS containing 0.5% bovine serum albumin (BSA), incubated with monoclonal mouse anti-HA antibody for 1 h at 4 ℃ and then with FITC-conjugated goat anti-mouse IgG for 30 min at 4 ℃, and resuspended in 500 µL of sterile PBS. The resulting *L. lactis*/pNZ8148-Spax-HA cells were subjected to immunofluorescence assay and FACS analysis, respectively.

### Quantification of HA protein expressed by *L. lactis*/pNZ8148-Spax-HA

The expression of HA protein by *L. lactis*/pNZ8148-Spax-HA was determined by indirect ELISA [29]. Briefly, 10^12^ colony forming units (CFUs) of *L. lactis*/pNZ8148-Spax-HA pellets were resuspended in 100 μL of a monoclonal mouse anti-HA antibody (0, 10, 25, 50, 75, 100, 125, 150, 175, and 200 μg/mL) in PBS containing 2% BSA, incubated at room temperature for 2 h, and incubated with goat anti-mouse IgG antibody conjugated to horseradish peroxidase (1 mg/mL) (Sigma-Aldrich Corporation, St. Louis, MO, USA) at room temperature for 1 h. The cells were then washed with sterile PBS and resuspended in 100 μL of HRP substrate 3,3′,5,5′-tetramethylbenzidine (TMB) (Sigma-Aldrich Corporation, St. Louis, MO, USA) in the dark for 25 min, and 100 μL of 2 mol/L H_2_SO_4_ was then added to stop the reaction. The OD_450nm_ value of the supernatant was measured using a microplate reader. *L. lactis*/pNZ8148-Spax was used as a negative control.

### Animals, immunization, sample collection and virus challenge

Specific pathogen-free (SPF) white Leghorn chickens (aged 7 days) were purchased from the Veterinary Research Academy of Agricultural Sciences of Jiangxi Province (Jiangxi, China) and housed in ventilated cages (five chickens per cage). The chickens were administered pelleted feed and sterile water and maintained in an SPF environment.

Three groups of 30 chickens each vaccinated orally with 2 mL of sterile PBS, 10^12^ CFU of *L. lactis*/pNZ8148-Spax or 10^12^ CFU of *L. lactis*/pNZ8148-Spax-HA, respectively. Prime immunization was performed at day 0, 1, and 2 and boosted at day 17, 18, and 19. PBS and *L.* lactis/pNZ8148-Spax cells were used as controls.

At days 15 and 34 after the initial vaccination, blood samples were collected from the wing vein. Sera were separated by centrifugation of the blood at 2000×g for 10 min and stored at − 20 °C until use. Meanwhile, the intestines were isolated from the vaccinated chickens (n = 3 per group at day 15, n = 3 per group at day 34) and washed with 500 µL of sterile PBS. Feces were also collected, resuspended in 500 µL of PBS and stored at − 20 °C until use.

Two weeks after the final vaccination, all the vaccinated chickens (n = 24 per group) were transferred into an animal biosafety level-3 (BSL-3) containment facility. Slight ether narcosis-anaesthetized chickens were intranasally infected with 20 µL of 5 × 50% lethal dose (5 × LD_50_) of HPAI H5N1 virus strains belonging to clade 1 (A/Vietnam/1203/2004, VN1203), clade 2.3 (A/Anhui/1/2005, Anhui) or clade 8 (A/chicken/Henan/12/2004, Henan).

Three chickens in each group were sacrificed at day 3 post challenge to check the virus titres in the lungs, as described previously [30]. Briefly, tenfold dilutions of lung homogenate supernatants were mixed with MEM containing trypsin, and reach up to 100 µL. Dilutions were added with 100 µL of Madin-Darby Canine Kidney (MDCK) cells at 2.5 × 10^6^ cells/mL, and incubated overnight at 37 °C. After 24 h incubation, 50 µL of 0.5% chicken red blood cells (CRBCs) was added, and then incubated for 1 h at room temperature and recorded hemagglutination afterwards to determine 50% tissue culture infective dose (TCID50). The other five chickens remaining in each group were used for survival records. The chickens were monitored every alternate day at a fixed time point to record their weight loss and survival. The humane endpoint of the challenge studies was a body weight loss exceeding 25% relative to the weight at the time of challenge inoculation. After final monitoring, all the surviving chickens were euthanized by CO_2_ inhalation for 5 min.

All animal immunizations were performed at a BSL-2 facility, and the virus challenge experiments were strictly performed in BSL-3 containment facilities, complied with the Guidelines for the Use and Care of Experimental Animals and were approved by the Institute Animal Care and Use Committee of Nanchang University.

### Determination of antibody responses by ELISA

Total serum antibody (IgY) and secretory IgA (sIgA) in the intestinal washes and feces were determined by ELISA using recombinant HA protein (2 µg/mL) from A/Vietnam/1203/2004 as a coating antigen as described previously [25]. In a brief, pNPP phosphatase substrate (MP Biomedicals, USA) was used for determining IgY titres. The OD value was measured at 405 nm using using an ELISA plate reader (Bio-Tek, USA). Furthermore, the intestinal washes and feces were used for determining sIgA using indirect ELISA. 3,3′,5,5′-Tetramethylbenzidine (TMB) was used as a substrate, and OD values were measured at 450 nm using an ELISA plate reader (Bio-Tek, USA). The IgY or sIgA titre was determined as the lowest dilution with an OD greater than the mean OD of the naïve controls plus two standard deviations.

### Neutralization assay

The neutralization activity of serum against different H5N1 viruses was assessed as described previously [25]. Briefly, receptor-destroying enzyme (RDE)-treated sera (n = 24 chickens per group) were serially diluted (twofold) and incubated with 100 TCID50 of viruses belonging to clade 1 (A/Vietnam/1203/2004, VN1203), clade 2.3 (A/Anhui/1/2005, Anhui) or clade 8 (A/chicken/Henan/12/2004, Henan) for 1 h at room temperature and plated in duplicate in a 96-well plate with MDCK cells. The neutralizing titre was assessed as the highest antibody dilution for which no cytopathic effect was observed by light microscopy.

### Statistical analysis

The statistical significance of the differences was assessed by Student’s *t* test and one-way ANOVA with multiple comparisons. Significant differences in the survival curves were determined by log-rank analysis. A *p* value less than 0.05 was considered to indicate statistical significance.

## Results

### Characterization of HA protein expressed on the surface of *L. lactis*

*L. lactis*/pNZ8148-Spax-HA was generated by fusing the HA gene of A/Vietnam/1203/2004 (clade 1) lacking a signal peptide and a transmembrane domain with Spax via a GS linker (Fig. 1a). This Spax was previously shown to be serve as effective anchor protein for display [26]. The expression of the HA protein was detected by Western blot analysis. As expected, a specific Spax-HA band was observed at the expected size (approximately 120 kDa) (Fig. 1b, Lane 2).

We then performed **i**mmunofluorescence assays and a flow cytometry analysis to determine the efficiency of the display of HA protein on the surface of *L. lactis*. *L. lactis*/pNZ8148-Spax-HA was incubated with mouse anti-HA monoclonal antibody for direct labelling. Compared with the *L. lactis*/pNZ8148-Spax control, positive fluorescence signals were observed with *L. lactis*/pNZ8148-Spax-HA (Fig. 1c, d)*.* Collectively, these results demonstrated that HA protein was located on the surface of *L. lactis*.

### Quantification of HA protein on the surface of *L. lactis*

Furthermore, as shown in Fig. 1e, When the concentration of antibody was increased beyond 125 μg/mL, the OD_450nm_ value was stable suggesting that 10^12^ CFUs of *L. lactis*/pNZ8148-Spax-HA expressed 125 μg of HA protein on the *L. lactis* surface.

### Antibody responses induced by *L. lactis*/pNZ8148-Spax-HA

**Fig. 2:**
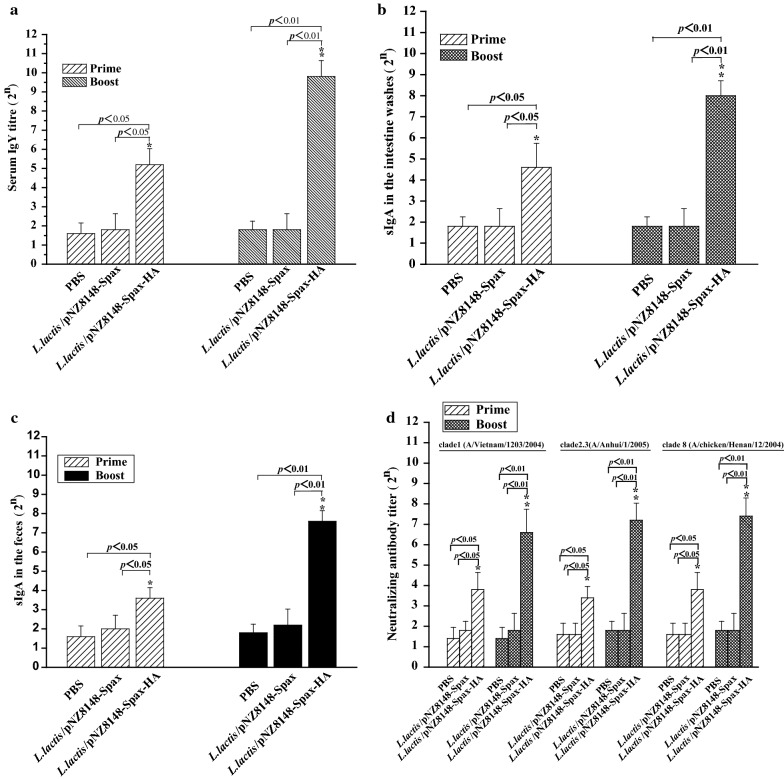
Determination of the antibody responses elicited by *L. lactis*/pNZ8148-Spax-HA in chickens. Sera, feces and intestine washes were collected from chickens vaccinated orally with PBS, *L. lactis*/pNZ8148-Spax or *L. lactis*/pNZ8148-Spax-HA. **a** HA-specific IgY antibody responses in sera. **b** HA-specific sIgA antibody responses in the intestine washes. **c** HA-specific sIgA antibody responses in the feces. **d** Neutralizing antibody titres. The data are represented as the means ± SDs, and the asterisks indicate significant differences compared with the PBS- and *L. lactis*/pNZ8148-Spax-vaccinated controls (*p* ˂ 0.05)

The HA-specific antibody responses were determined by ELISA. At day 15, the sera IgY titres from chickens (n = 24 per group) vaccinated orally with *L. lactis*/pNZ8148-Spax-HA showed a slightly significant difference compared with that found in the control chickens vaccinated with PBS and the *L. lactis*/pNZ8148-Spax control. However, a highly significant increase was detected in the *L. lactis*/pNZ8148-Spax-HA group at day 34, whereas after the boost immunization, no significant changes were found in the groups vaccinated with PBS or *L. lactis*/pNZ8148-Spax (Fig. 2a). Similarly, a significant level of mucosal sIgA antibody was detected in the intestine washes and feces of chickens (n = 3 per group at day 15, n = 3 per group at day 34) vaccinated orally with *L. lactis*/pNZ8148-Spax-HA (Fig. 2b, c). These results revealed that a prime-boost regimen of *L. lactis*/pNZ8148-Spax-HA could induce significant humoral and mucosal immune responses in vaccinated chickens.

### Neutralization assay

Due to frequent mutation of the H5N1 virus, it is important to determine whether vaccine candidates elicit neutralization antibodies against different H5N1 clades. We thus performed neutralization tests to examine the ability of *L. lactis*/pNZ8148-Spax-HA to generate cross-neutralizing antibody responses against different H5N1 viruses. As shown in Fig. 2d, cross-neutralizing antibodies were elicited in the *L. lactis*/pNZ8148-Spax-HA group, and this finding was similar with the HA-specific IgY response. In contrast, no significant cross-neutralizing antibodies were detected in the *L. lactis*/pNZ8148-Spax control group. These data were consistent with the ELISA results: chickens vaccinated orally with *L. lactis*/pNZ8148-Spax-HA using the prime/boost regimen could produce a higher HA-specific IgY antibody and elicit neutralization activities, which might contribute to the prevention of H5N1 clade 1 (A/Vietnam/1203/2004, VN1203), clade 2.3 (A/Anhui/1/2005, Anhui) and clade 8 (A/chicken/Henan/12/2004, Henan) infection.

### Cross-protection against H5N1 virus infection

**Fig. 3:**
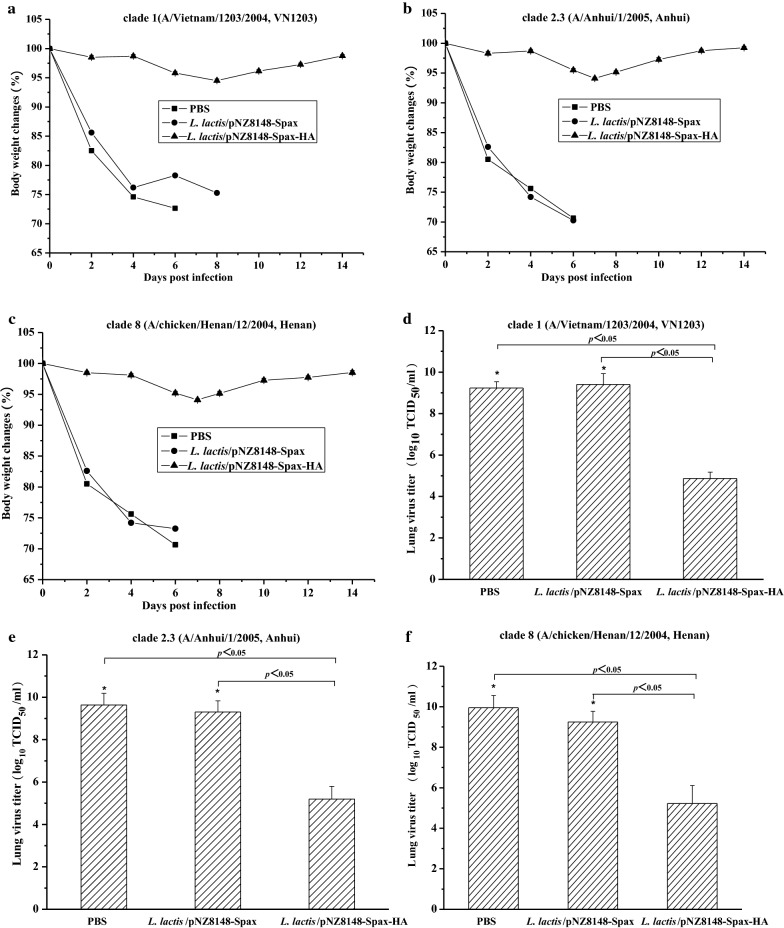

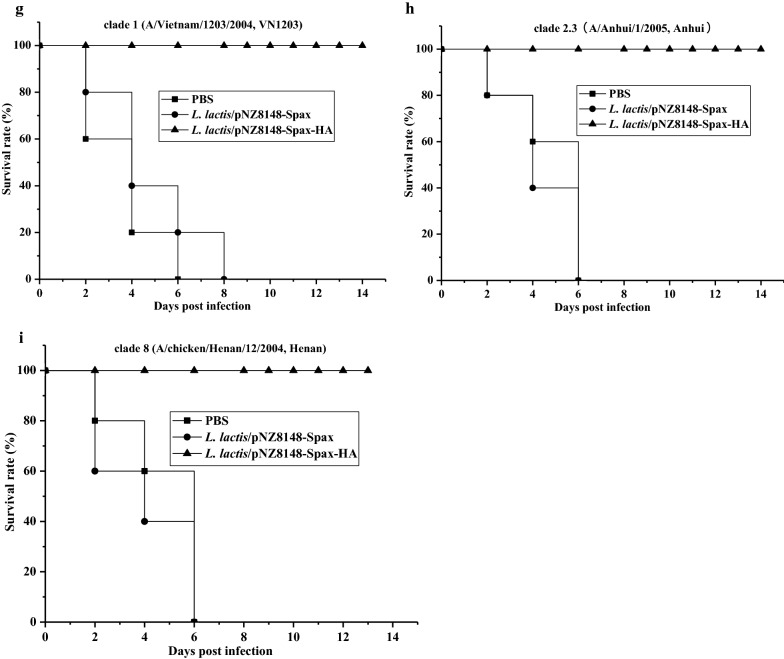
Cross-clade protection of chickens against lethal challenge with different H5N1 viruses. Two weeks after the last immunization, the chickens (n = 24/group) were intranasally infected with 20 µL of 5 × LD_50_ of HPAI H5N1 virus strains belonging to clade 1 (A/Vietnam/1203/2004) (**a**, **d**, **g**); clade 2.3 (A/Anhui/1/2005, Anhui) (**b**, **e**, **h**) or clade 8 (A/chicken/Henan/12/2004) (**c**, **f**, **i**). The results are expressed in terms of the percent body weight (**a**–**c**), lung virus titre (**d**–**f**) and percent survival (**g**–**i**). The chickens were monitored throughout a 14-day observation period (n = 5/group). The data on the virus titres in the lung are presented as the means ± SDs. The asterisks indicate significant differences compared with the PBS- and *L. lactis*/pNZ8148-Spax-vaccinated controls (*p* ˂ 0.05)

To further support the cross-protective potential of the *L. lactis*/pNZ8148-Spax-HA vaccine, vaccinated chickens (n = 24 per group) were inoculated intranasally with 20 µL of 5 × LD_50_ of a H5N1 virus belonging to clade 1 (A/Vietnam/1203/2004, VN1203), clade 2.3 (A/Anhui/1/05, Anhui) or clade 8 (A/chicken/Henan/12/2004, Henan), respectively, and monitored for 14 days. The control groups that received PBS or *L. lactis*/pNZ8148-Spax showed clinical signs of severe disease, a significant body weight loss, and a higher lung virus titre after virus infection and died within 8 days after the lethal challenge (Fig. 3). In contrast, the chickens vaccinated orally with *L. lactis*/pNZ8148-Spax-HA survived throughout the study period and had recovered within 14 days after challenge, and no significant weight loss or apparent illness symptoms were noted after virus challenge (Fig. 3). A lower virus titre in the lung was observed in the *L. lactis*/pNZ8148-Spax-HA group (Fig. 3). Taken together, the results from the virus challenge experiments provided reliable evidence showing that the *L. lactis*/pNZ8148-Spax-HA vaccine candidate conferred cross-clade protection against divergent H5N1 clades in the chicken model.

## Discussion

Due to the frequent occurrence of antigenic changes in HPAI H5N1 viruses, there is a risk for an epidemic or potential pandemic of these viruses in poultry. A well-matched H5N1 vaccine is likely an effective measure for fighting a potential H5N1 panzootic. In this study, we generated a well-characterized *L. lactis*/pNZ8148-Spax-HA vaccine candidate based on the *L. lactis* display platform, which was previously found to exhibit several advantages over other vaccine approaches, such as the easy realization of genetic modifications, an efficient and cost-effective manufacturing process, a mucosal delivery route and a proven safety and immunogenicity profile [31].

To define a more optimized vaccine approach, we investigated the immunogenicity of unadjuvanted *L. lactis*/pNZ8148-Spax-HA administered orally in the chicken model. It is generally accepted that trimeric HA exhibits strong immunogenicity, and previous studies have revealed the promising finding that chickens injected with adjuvanted subunit vaccines consisting of trimeric H5N1 HA exhibit high levels of cross-neutralizing antibodies against H5N1 clades 1 and 2 [32, 33]. Furthermore, Oral administration of recombinant *L. lactis* expressing functional influenza NA or M2e proteins elicites effective mucosal and systemic immune responses without the use of adjuvant in the chicken model, and protects MDCK cells against A/PR/8/34 (H1N1) virus challenge [28], however, the cross-protective efficacy of *L. lactis/*pNZ-NA or *L. lactis/*pNZ-4xM2e in chickens against virus challenge is not yet investigaged. To further assess the cross-protection of *L. lactis* vectored vaccine in the chicken model, we demonstrated the unadjuvanted *L. lactis*/pNZ8148-Spax-HA in which the HA gene lacks the signal and trimerization sequence provided convincing cross-clade protection following prime/boost oral vaccination. Furthermore, the *L. lactis* vectored H5N1 vaccine candidate has advantages compared with the currently used influenza virus vaccines, including easy generation of the vectors and the ability to produce vaccines in cell lines that have already been approved for the manufacturing of human vaccines [34]. In addition, *L. lactis* vaccines are more effective and safer compared with viral vector-based approaches and do not require adjuvants [35]. Thus, the *L. lactis* display platform would have a beneficial effect on the manufacturing of influenza vaccines.

An anchor protein plays an important role in *L. lactis* display-based vaccines. Our previous studies showed that pgsA and Spax could be used as anchor proteins for antigen display [26, 36]. *L. lactis*/pNZ8110-pgsA-HA1 adjuvanted with CTB provides immune protection against homologous H5N1 virus in a mouse model [36]. Additionally, unadjuvanted *L. lactis*/pNZ8110-pgsA-NA and unadjuvanted *L. lactis*/pNZ8008-Spax-HA2 could protect mice from homologous and heterologous virus infection [26, 37]. The comparisons of the display efficiency of *L. lactis*-displayed influenza vaccines obtained with various anchor proteins revealed that Spax was superior to pgsA. Thus, in our present study, Spax was selected for the design of *L. lactis*-displayed H5N1 HA, and unadjuvanted *L. lactis*/pNZ8148-Spax-HA conferred cross-clade protection against different H5N1 viruses in the chicken model. Based on these findings, the *L. lactis* display platform using Spax will contribute to improving the feasibility of developing influenza H5N1 vaccines for poultry that confer protection against different H5N1 clades.

The rapid evolution of new sublineages of influenza A/H5N1 virus poses a great threat to poultry health [38]. A major obstacle in vaccine development against influenza H5N1 virus infection is the rapid evolution of the genetic diversity of these viruses [39]. As a result and given the limitations of the currently approved vaccines for H5N1 viruses in terms of their production timelines and their ability to induce cross-clade protective immune responses, newer vaccine approaches for panzootic preparedness against these viruses are needed [40]. The development of a simple vaccine that provides broad protection against influenza H5N1 is of high priority for preparing the poultry industry against a future influenza panzootic. This study clearly demonstrated that unadjuvanted *L. lactis*/pNZ8148-Spax-HA could serve as an alternative approach that is currently showing promise for the development of a H5N1 cross-clade vaccine for the mass vaccination of poultry. Overall, the *L. lactis* display platform might constitute a new strategy for the development of a universal flu vaccine for poultry and humans.

## Conclusions

The findings obtained in this study suggest that the unadjuvanted *L. lactis* display platform can be used to an alternative H5N1 cross-clade vaccine candidate and can overcome the bottleneck of the current manufacturing process because it constitutes a flexible and high-output system for the safe, effective and low-cost production of an H5N1 vaccine for poultry during an influenza H5N1 panzootic.

## Data Availability

The datasets generated and analysed in the current study are available from the corresponding author upon reasonable request.
